# Administration of Protocatechuic Acid Reduces Traumatic Brain Injury-Induced Neuronal Death

**DOI:** 10.3390/ijms18122510

**Published:** 2017-11-23

**Authors:** Sang Hwon Lee, Bo Young Choi, Song Hee Lee, A. Ra Kho, Jeong Hyun Jeong, Dae Ki Hong, Sang Won Suh

**Affiliations:** Department of Physiology, College of Medicine, Hallym University, Chuncheon 24252, Korea; bluesea3616@naver.com (S.H.L.); bychoi@hallym.ac.kr (B.Y.C.); sshlee@hallym.ac.kr (S.H.L.); rnlduadkfk136@hallym.ac.kr (A.R.K.); jd1422@hanmail.net (J.H.J.); zxnm01220@gmail.com (D.K.H.)

**Keywords:** traumatic brain injury, protocatechuic acid, neuron death, oxidative injury, microglial activation

## Abstract

Protocatechuic acid (PCA) was first purified from green tea and has shown numerous biological activities, including anti-apoptotic, anti-inflammatory, and anti-atherosclerotic effects. The effect of PCA on traumatic brain injury (TBI)-induced neuronal death has not previously been evaluated. TBI is defined as damage to the brain resulting from external mechanical force, such as rapid acceleration or deceleration, impact, blast waves, or penetration by a projectile. TBI causes neuronal death in the hippocampus and cerebral cortex. The present study aimed to evaluate the therapeutic potential of PCA on TBI-induced neuronal death. Here, TBI was induced by a controlled cortical impact model using rats. PCA (30 mg/kg) was injected into the intraperitoneal (ip) space immediately after TBI. Neuronal death was evaluated with Fluoro Jade-B (FJB) staining at 24 h after TBI. Oxidative injury was detected by 4-hydroxy-2-nonenal (4HNE), glutathione (GSH) concentration was analyzed by glutathione adduct with *N*-ethylmaleimide (GS-NEM) staining at 24 h after TBI, and microglial activation in the hippocampus was detected by CD11b immunohistochemistry at one week after TBI. We found that the proportion of degenerating neurons, oxidative injury, GSH depletion, and microglia activation in the hippocampus and cortex were all reduced by PCA treatment following TBI. Therefore, our study suggests that PCA may have therapeutic potential in preventing TBI-induced neuronal death.

## 1. Introduction

Traumatic brain injury (TBI) is an injury to the central nervous system that temporarily or permanently impairs brain function due to the effects of blunt or mechanical force trauma. TBI is one of the leading causes of death and disability worldwide for children and young adults, owing to a multitude of factors, including car accidents, sports, or violence. Even following mild TBI, brain function is often diminished or temporarily impaired [1]. Thus, TBI can cause serious problems in the social, cognitive, physical, behavioral, and emotional domains. TBI is defined as injury to the central nervous system (CNS) resulting from mechanical force, including impact, rapid acceleration, or penetration by projectiles [2]. Primary TBI is caused by tissue laceration, blood vessel rupture, or neuron/glia compression or tearing. This primary injury induces several cascading degenerative processes occurring from immediately after the TBI to several days after the TBI. Currently, TBI outcomes have been improved with newly developed clinical management practices, such as the use of antibiotics or control of brain edema [3]. However, many survivors from TBI still display motor dysfunction and permanent cognitive impairment, and there are currently no effective therapeutic tools for preventing chronic motor dysfunction and cognitive problems, even after the acute TBI symptoms have been corrected. Increasing evidence suggests that reactive oxygen species (ROS) are involved in the pathophysiology of neuronal injuries, including TBI [4,5,6,7,8,9]. Furthermore, several free radical scavengers have been demonstrated to improve brain function after TBI [10,11]. The production of reactive oxygen species (ROS) have been demonstrated in traumatic [12,13,14] as well as excitotoxic brain injury [15,16]. However, ROS may also promote the synaptic release of glutamate [17]. This suggests that glutamate release and ROS production are involved and cooperate in a cascade of events that could cause neuronal death. The close relationship between TBI and oxidative stress has generated considerable interest in the development of antioxidant agents to prevent the deleterious consequences of oxidative insults after TBI.

This study aimed to evaluate the therapeutic potential of protocatechuic acid (PCA) on TBI-induced neuronal death. PCA, one of the main metabolites of complex polyphenols, is a dihydroxybenzoic acid naturally found in green tea, and has strong free radical scavenging effects. PCA displays numerous biological activities, including anti-oxidant, anti-atherosclerotic, anti-apoptotic, anti-inflammatory, and neuroprotective activity [18,19,20,21,22,23,24,25,26,27]. PCA was reported to be significantly more concentrated and with longer retention in plasma than other polyphenol compounds, and can pass through the blood brain barrier (BBB) easily [28]. PCA has been shown to reduce reactive oxygen species (ROS), but its efficacy for ameliorating neuronal death after TBI has not been evaluated. In this study, we found that TBI-induced oxidative injury, glutathione (GSH) depletion, microglial activation, and neuronal death were prevented by the administration of PCA. Therefore, these results suggest that post-treatment with PCA may have a high therapeutic potential for treating TBI-induced neuronal death.

## 2. Results

### 2.1. Protocatechuic Acid (PCA) Decreases the Number of Degenerating Neurons after Traumatic Brain Injury (TBI)

**Figure 1:**
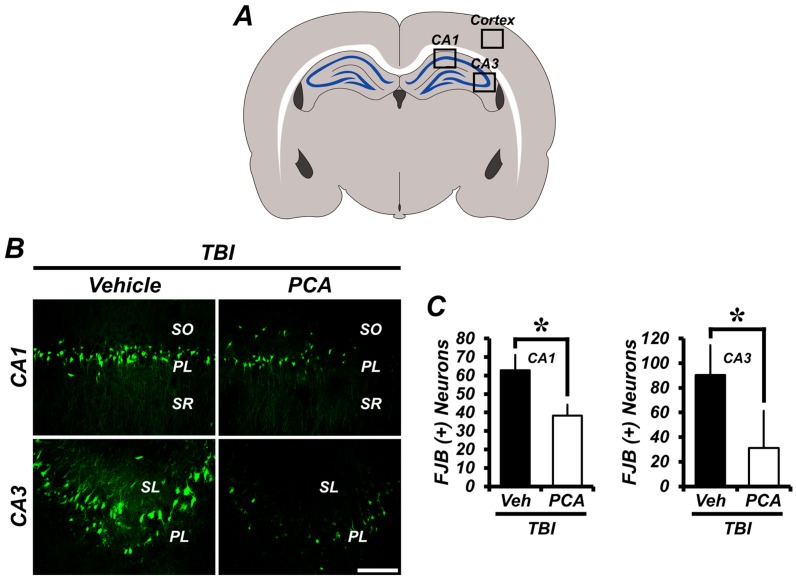

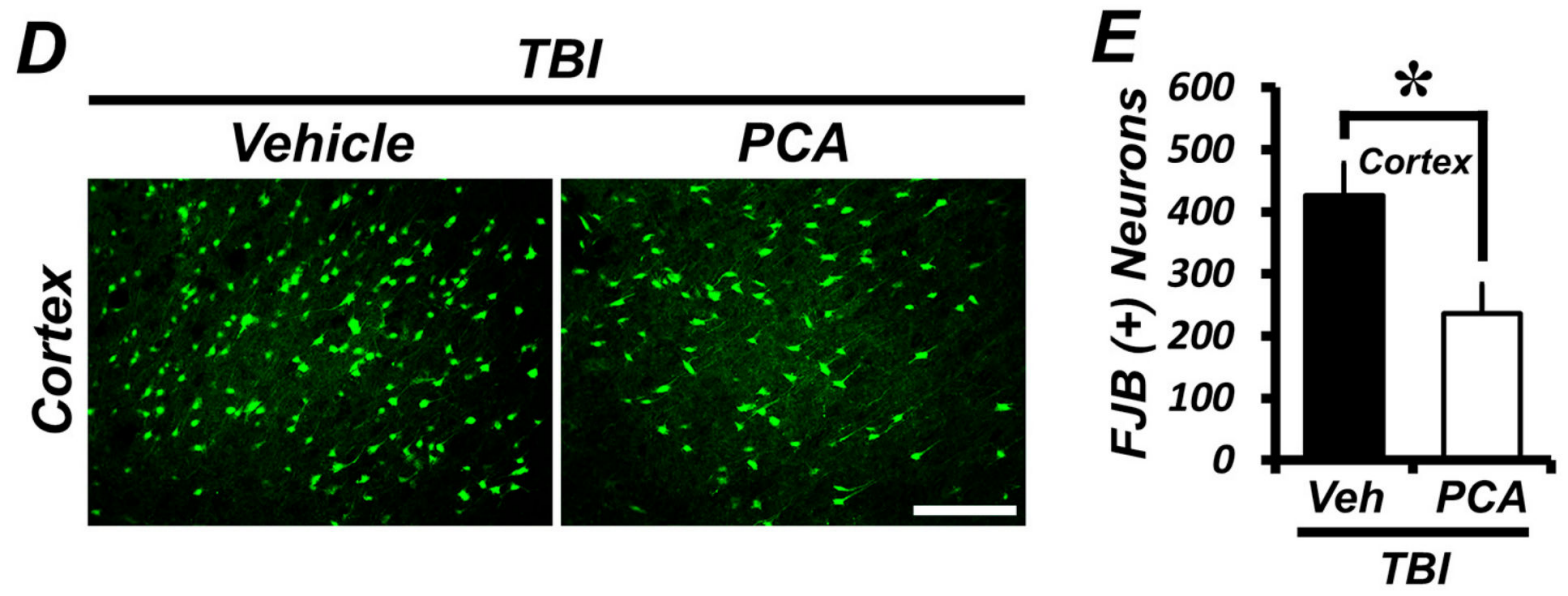
Protocatechuic acid (PCA) reduces the number of degenerating neurons after traumatic brain injury (TBI). Neuronal death was examined by Fluoro-Jade B (FJB) staining. Thus, FJB positive neurons were not detected in the sham-operated groups. (**A**) A schematic drawing indicates quantified area in the hippocampus and cerebral cortex. (**B**,**D**) Fluorescence images represent the relative degree of neuronal death in the hippocampus (**B**) and cortex (**D**) at 24 h after TBI. PCA administration reduced the number of FJB (+) neurons in the hippocampus and cortex when compared to the vehicle-treated group. Scale bar = 100 µm. (**C**,**E**) The bar graph shows the number of degenerating neurons in the hippocampus (**C**) and cortex (**E**). Data are mean ± SEM, and *n* = 10 from each TBI group. * Significantly different from vehicle-treated group, *p* < 0.05. Veh = Vehicle; SR = Stratum Radiatum; PL = Pyramidal Layer; SO = Stratum Oriens of hippocampus cornu ammonis 1 (CA1) and CA3 area.

To test whether PCA treatment showed neuroprotective effects after TBI, rats were sacrificed 24 h after insult, with or without PCA injection. Neuronal injury was evaluated by Fluoro-Jade B (FJB) staining. There were no FJB (+) neurons in the sham-operated group. After TBI, FJB (+) neurons were observed in cornu ammonis 1 (CA1), CA3, and the cortex area. Compared with the vehicle treated control group, the PCA-treated (30 mg/kg) group showed a reduced number of FJB (+) neurons. Degenerating neurons in the PCA-treated group decreased by 38%, 67%, and 43%, when compared to the vehicle-treated group in CA1, CA3, and cortex area, respectively (Figure 1).

### 2.2. PCA Decreases TBI-Induced Oxidative Injury in the Hippocampus and Cortex

**Figure 2:**
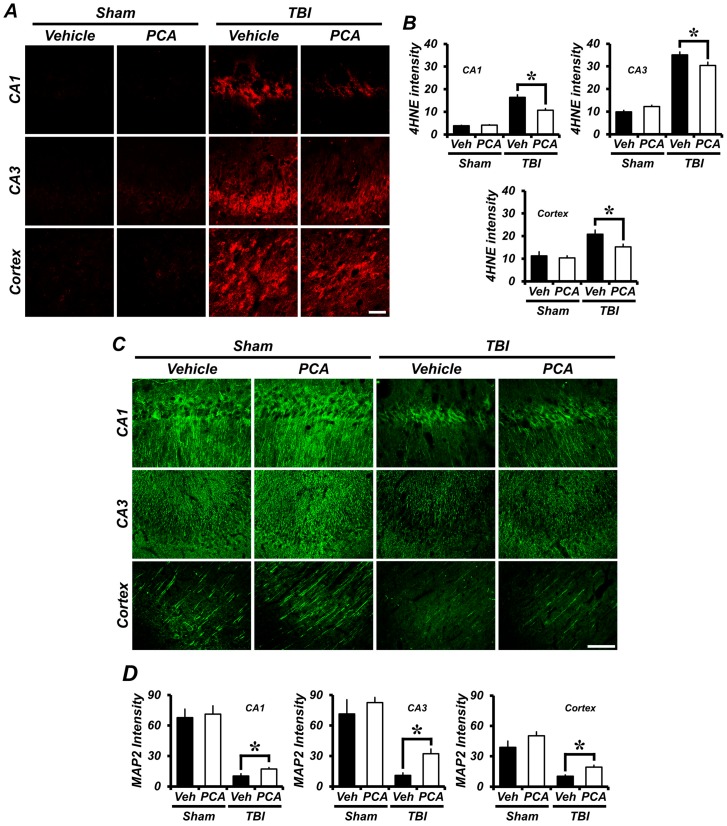
PCA reduces TBI-induced oxidative injury. (**A**) Neuronal oxidative injury was examined by 4-hydroxy-2-nonenal (4HNE) (**red color**) staining. Fluorescence images represent the degree of oxidative injury present in the hippocampus and cortex at 24 h after TBI. PCA administration reduces the intensity of oxidative injury in the hippocampus and cortex when compared to the vehicle-treated group. Scale bar indicates 50 µm. (**B**) The bar graph shows the 4HNE fluorescence intensity in the hippocampus and cortex. (**C**) Dendritic loss was examined by microtubule-associated protein 2 (MAP2) (**green color**) staining. Fluorescence images represent the results of MAP2 in the hippocampus and cortex at 24 h after TBI. PCA administration reduced the intensity of dendritic loss in the hippocampus and cortex when compared to the vehicle-treated group. Scale bar indicates 100 µm. (**D**) The bar graph shows the MAP2 fluorescence intensity in the hippocampus and cortex. Data are mean ± SEM, *n* = 5 from each sham group and *n* = 10 from each TBI group. * Significantly different from vehicle-treated group, *p* < 0.05.

To detect oxidative injury after TBI, we evaluated the oxidative injury using 4-hydroxy-2-nonenal (4HNE) staining. Rat brain samples were immunohistochemically stained with a 4HNE antibody at 24 h after TBI to reveal whether oxidative stress had occurred in the hippocampal and cortical neurons. As estimated in Figure 2B, quantification of oxidative injury in the TBI vehicle-treated group increased by 320%, 350%, and 200% in the CA1, CA3, and cortex, respectively, when compared to the sham vehicle-treated group. In contrast, the TBI PCA-treated group increased by only 130%, 260%, and 50% in the CA1, CA3, and cortex, respectively, when compared with the sham vehicle-treated group (Figure 2A,B).

### 2.3. PCA Decreases TBI-Induced Dendritic Damage in the Hippocampus and Cortex

To evaluate whether TBI induced changes in cortical dendritic structure, brains were histologically evaluated by microtubule-associated protein 2 (MAP2) immunostaining at 24 h after TBI. TBI-induced rats showed significantly low MAP2 immunoreactivity (IR) in the hippocampus and in the cortex when compared with PCA-treated rats, indicating a loss of dendrites. PCA administration reduced TBI-induced dendrite loss when compared to the vehicle-treated group (Figure 2C,D).

### 2.4. PCA Decreases TBI-Induced Glutathione (GSH) Depletion in the Hippocampus and Cortex

**Figure 3:**
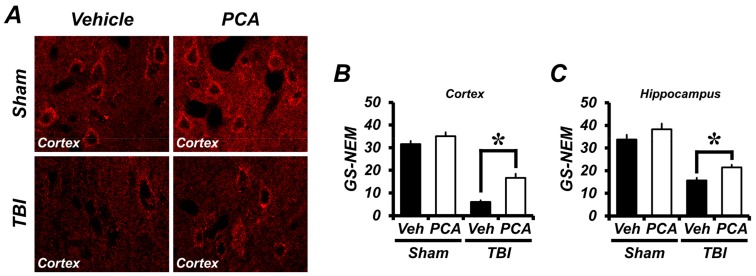
PCA reduces TBI-induced glutathione depletion in the hippocampus and cortex. (**A**) Fluorescence photomicrographs of brain sections stained with glutathione adduct with *N*-ethylmaleimide (GS-NEM) antibody. Sham-operated brain sections contain mostly GS-NEM in the hippocampal neurons and cortical neurons. Images show several GS-NEM negative neurons in the cortex area at one week after TBI. PCA administration increases the intensity of glutathione level in the hippocampus and cortex. Scale bar represents 20 µm. (**B**,**C**) The bar graph shows the quantified GS-NEM fluorescence intensity in the cortex (**B**) and hippocampus (**C**). The GS-NEM fluorescence intensity was found to be significantly different between the vehicle and PCA treated group in the hippocampus and cortex area. Data are mean ± SEM, *n* = 5 from each sham group and *n* = 10 from each TBI group. * Significantly different from vehicle-treated group, *p* < 0.05.

To evaluate the changes to GSH content in the hippocampal pyramidal neurons, brain sections were stained with glutathione adduct with *N*-ethylmaleimide (GS-NEM). Levels of GSH fluorescence in the hippocampal neurons and cortical neurons were analyzed using ImageJ. GSH concentrations in the hippocampus and cortex were significantly decreased by TBI. However, GSH concentrations in the hippocampal and cortical neurons were 146% to 360% higher, respectively, in the PCA-treated group when compared to the vehicle-treated group after TBI (Figure 3).

### 2.5. PCA Decreases TBI-Induced Microglial and Macrophage Activation

**Figure 4:**
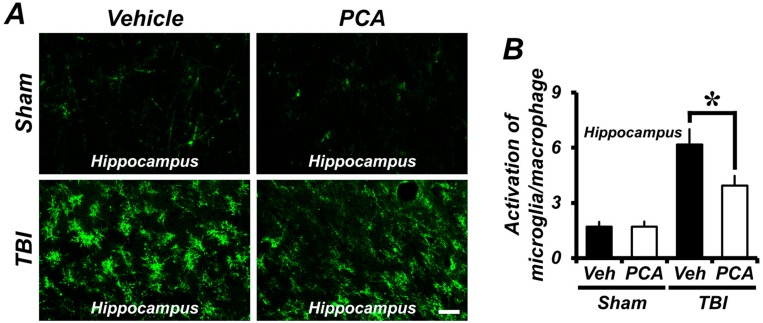

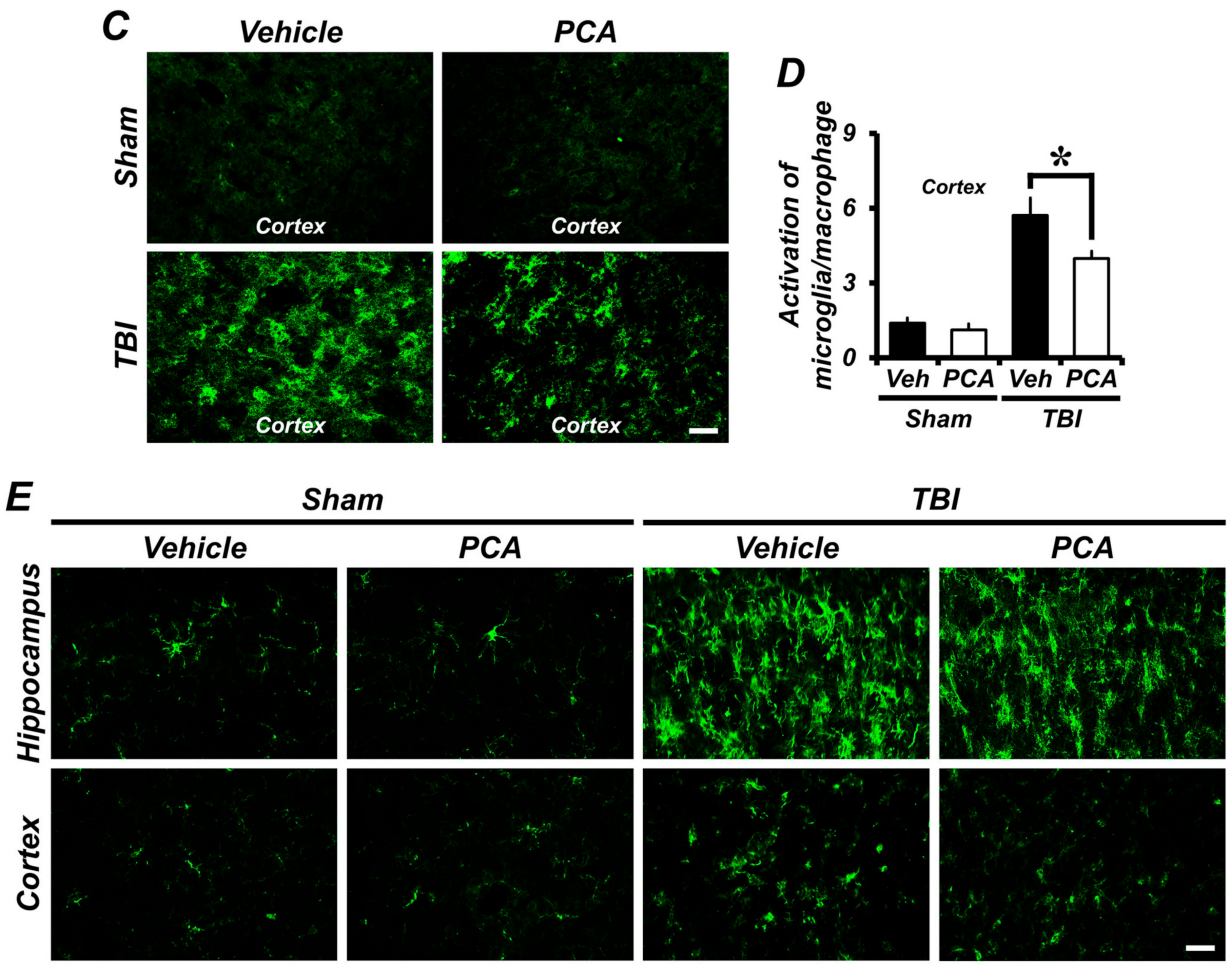
PCA reduces TBI-induced microglia and macrophage activation. (**A**,**C**) Representative images showed CD11b immunoreactive cells in the hippocampus (**A**) and cortex (**C**). The activation of CD11b-positive microglia and macrophage occurred at one week after TBI. PCA administration reduced the activation of CD11b-positive microglia and macrophage in the hippocampus, compared to the vehicle-treated group. Scale bar = 20 µm. (**B**,**D**) The bar graphs represent the grade of CD11b-positive microglia/macrophage activation in the hippocampus (**B**) and cortex (**D**). Data are mean ± SEM, *n* = 5 from each sham group and *n* = 10 from each TBI group. * Significantly different from vehicle-treated group, *p* < 0.05. (**E**) Fluorescence photomicrographs represented Iba1 immunoreactive cells in the hippocampus and cortex. Sham-operated groups showed the resting state of Iba1-positive microglia and macrophage. There was no difference in the Iba1 immunoreactivity (IR) between vehicle- and PCA-treated group. However, TBI caused significant increase of Iba1 IR in the hippocampus and cortex. PCA treatment reduced the activation of Ionized calcium binding adaptor molecule 1 (Iba1)-positive microglia and macrophage after TBI, compared to the vehicle-treated group.

To test the effects of PCA on TBI-induced microglia activation, brain sections were prepared from sham-operated or one week post TBI-induced animals. The activation of microglial and macrophage was analyzed by CD11b immunoreactive cells and their morphology. TBI-induced rats showed significantly increased CD11b IR in the hippocampus when compared with the sham-operated rats. However, CD11b IR in PCA-treated group was decreased by 34% when compared to the vehicle-treated group after TBI (Figure 4A–D). In addition, we also checked Ionized calcium binding adaptor molecule 1 (Iba1) IR, a macrophage/microglia-specific calcium-binding protein. Sham-operated rats showed the morphology of resting microglia and macrophage in the hippocampus and cortex. After TBI, Iba1 IR were remarkably increased in vehicle-treated group, but PCA-treated group showed significant reduction of the Iba1 IR compared to the vehicle-treated group (Figure 4E). Therefore, these results suggest that TBI-induced microglia and macrophage activation were reduced by PCA treatment.

### 2.6. PCA Prevents Delayed Neuronal Death after TBI

**Figure 5:**
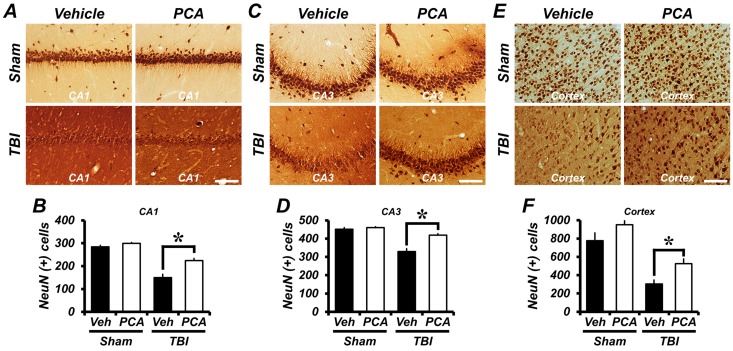
PCA prevents delayed neuronal death after TBI. (**A**,**C**,**E**) Representative images show the live neurons in the CA1 (**A**), CA3 (**C**), and cortex (**E**) at one week after Sham operation or TBI surgery. Sham-operated groups did not show neuronal loss. However, the number of NeuN (+) neurons was significantly decreased in the CA1, CA3, and cortex after TBI. In addition, PCA-treated TBI group showed more NeuN (+) neurons when compared to the vehicle-treated TBI group. (**B**,**D**,**F**) The bar graphs show the quantified number of NeuN (+) neurons in the CA1 (**B**), CA3 (**D**), and cortex (**F**) after TBI. Data are mean ± SEM, *n* = 5 from each sham group and *n* = 10 from each TBI group. * Significantly different from vehicle-treated group, *p* < 0.05.

To test whether PCA had neuroprotective effects, NeuN staining was performed at one week after TBI. As estimated in Figure 5, the number of NeuN (+) neurons in the vehicle-treated TBI group was decreased by 50%, 29%, and 63% from the CA1, CA3, and cortex, respectively, when compared to the vehicle-treated sham group. However, the number of NeuN (+) neurons in the PCA-treated TBI group was significantly increased by 24%, 37%, and 48%, when compared to the TBI-vehicle treated group in the CA1, CA3, and the cortex, respectively. These results suggested that TBI-induced delayed neuronal loss was prevented by PCA treatment (Figure 5).

## 3. Discussion

In this study, we evaluated the therapeutic effects of PCA on TBI-induced brain damage, by using brain sections to test whether PCA had any beneficial effects on TBI-induced oxidative injury, GSH restoration, microglia activation, and finally, neuronal death. TBI directly damaged CA1 and CA3 of the hippocampus and cerebral cortex. Thus, we selected this region, as our TBI model directly affected these regions. The present study demonstrated that PCA showed protective effects on TBI-induced neuronal cell death.

PCA has been recognized as a cytoprotective agent through its anti-apoptotic, anti-oxidative, and anti-inflammatory effects that have been demonstrated in previous studies [23,29,30,31]. Antioxidative effects of PCA is 10-fold higher than α-tocopherol. Polyphenols have been associated with beneficial roles for human health including cancer, diabetes mellitus, cerebrovascular diseases. However, whether PCA has any therapeutic effects on TBI-induced neuronal death has not been tested previously. Since PCA has low cost, more potential than other antioxidants and without adverse side effects, the present study tested it as a potential therapeutic candidate for preventing TBI-induced neuron death.

Oxidative damage has been implicated in a variety of cell death signaling pathways [32], and is thought to play an important role in the pathogenesis of degenerative neuronal diseases [33,34]. Therefore, we conducted 4HNE staining to evaluate oxidative stress in the hippocampus. TBI increased oxidative stress in the hippocampus and cerebral cortex, and we found that TBI-induced oxidative injury was reduced by PCA treatment.

To clarify, if the protective effect of PCA was associated with its anti-oxidative effects, we measured GSH concentration by GS-NEM immunohistochemical staining. GSH is capable of reducing injury to cellular components induced by ROS, including free radicals, lipid peroxides, and heavy metals [35]. Reduced GSH, a non-protein thiol compound, is involved in intracellular redox regulation, detoxification, immunity, and cell signaling [36]. Antioxidants, such as reduced GSH, offset the deleterious effects of ROS. Therefore, the recovery of neuronal GSH by PCA administration can decrease ROS production, which eventually protects neuronal death after TBI. In the present study, we found that TBI reduced neuronal glutathione concentrations in the hippocampus and cortex. However, PCA reversed this TBI-induced glutathione depletion in the hippocampus and cerebral cortex. Further research should be conducted into how PCA reverses TBI-induced GSH depletion.

Microglia and macrophages are the constitutive immune cells of the CNS, and have critical roles in the host innate immune response. Activated microglia and macrophages produce numerous proinflammatory cytokines, and play an important role in the pathogenesis of neurodegenerative diseases [37]. Damaged neurons can induce microglia and macrophage activation, and excessive microglia and macrophage activation can have deleterious effects on neuronal survival, since activated microglia release reactive oxygen, glutamate, extracellular proteases, and cytokines, which are toxic to neurons. In the present study, microglial and macrophage activation were detected by CD11b or Iba1 immunohistochemistry in the hippocampus and cortex at one week after TBI. TBI significantly increased microglial and macrophage activation in the hippocampus and cortex, which may have a large impact on hippocampal and cortical neuronal death by promoting the deleterious toxic functions described above. However, administration of PCA decreased microglial and macrophage activation in the hippocampus and cortex.

The present study includes the following limitations: (1) the TBI model used is not physiologically relevant, as the vast majority of TBI incidents do not involve penetration of the skull. (2) The administration of PCA immediately after TBI is also not realistic. Because most TBI patients do not receive treatment immediately following TBI. (3) Neuronal protection was only observed at 24 h and 7 days post-TBI. It would be interesting to explore neuronal protection after a more prolonged period with PCA administration. (4) No behavioral, cognitive, or motor assessments were performed in the present study. Exploring the effect of PCA after TBI on these aspects could be a future direction for research.

Despite these limitations, we found that PCA administration prevented neuronal loss after TBI through reduced oxidative damage, microglial activation, and glutathione depletion, which in aggregate prevented neuronal loss after TBI. Taken together, the present study suggests that PCA has therapeutic potential for the prevention of TBI-induced brain injury.

## 4. Materials and Methods

### 4.1. Experimental Animals

The animal handling procedures were conducted in accordance with the guidelines of the Institutional Animal Care and Use Committee of Hallym University (Protocol #Hallym-2016-17). Adult male Sprague-Dawley rats (SD-Rat) were used in this study (8 weeks, 270–300 g, DBL Co., Chungcheongbuk, Korea). The animals were housed in a constant humidity and temperature controlled environment (12 h light–12 h dark cycle, 55 ± 5%, 22 ± 2 °C); a Purina diet (Cargill Agri Purina Inc., Seongnam-si, Gyeonggi, Korea) and water were provided ad libitum. This protocol was written up in agreement with the ARRIVE (Animal Research: Reporting in Vivo Experiments) guidelines.

### 4.2. Controlled Cortical Impact Model for TBI

Rats were placed in a stereotaxic frame and anesthetized with 2% isoflurane and a 25:75 mixture of oxygen/nitrous oxide (David Kopf Instruments, Tujunga, CA, USA). The scalp and temporalis muscles were reflected, and a 5.0 mm diameter hole was drilled through the skull (4.5 mm Lambda to the Bregma and 2.8 mm lateral to the midline) [38,39,40,41]. TBI was performed using an electromagnetic (EM) controlled cortical impact device (Impact One TM Stereotaxic Impactor, Richmond, IL, USA). For the mechanical trauma, a 5 mm blunt steel impactor tip was retracted and positioned above the intact dura. The injury was triggered using the My Neuro Lab (Impact One^TM^ Stereotaxic Impactor, Richmond, IL, USA) controller at a strike velocity of 5.0 m/s, strike depth of 3.0 mm, and dwell time of 500 ms [42]. TBI induction was performed in only one side of the brain hemisphere, and only minor damage was applied in the opposite hemisphere. In the case of the sham-operated group, the skull was opened and treated with vehicle or PCA. All rats were maintained at a core temperature of 36–37.5 °C during and after surgery, until ambulatory.

### 4.3. PCA Administration

PCA was dissolved in normal saline (0.9% NaCl). Rats were injected with protocatechuic acid (PCA, 30 mg/kg, ip) once per day for one week, starting immediately after TBI. Control rats were injected with the same volume of normal saline. The non-TBI group also had PCA/saline or saline vehicle only. Animals were divided into four groups: (1) sham without PCA (saline only, *n* = 5); (2) sham with PCA (PCA only, *n* = 5); (3) TBI without PCA (TBI + saline, *n* = 10); and (4) TBI with PCA (TBI + PCA, *n* = 10).

### 4.4. Brain Section Groundwork

For detecting neuronal death, oxidative injury, and glutathione (GSH) concentrations, animals were sacrificed at 24 h after TBI. For detecting microglial activation in the hippocampus animals were sacrificed at one week after TBI. The animals were anesthetized with urethane (1.5 g/kg, ip), and 0.9% saline was perfused through the heart, and the brain was fixed with 4% paraformaldehyde (PFA). The brains were post-fixed in paraformaldehyde for 1 h. The brains were submersed in 30% sucrose for 2 days. Using a cryostat microtome, tissues were collected with a thickness of 30 µm.

### 4.5. Evaluation of Neuronal Death

Neuronal death was measured by FJB staining, as described by Hopkins and Schmued [43,44]. To begin staining, the brain sections (30 μm) were first placed on a gelatin coated slide (Pittsburgh, Fisher Scientific, PA, USA). The slides were immersed in 0.06% potassium permanganate for 15 min. The slides were then immersed in 0.001% FJB (Histo-Chem Inc., Jefferson, AR, USA) for 30 min. Next, brain sections were washed three times for 1 min in distilled water. Sections were dried and observed with a fluorescence microscope excitation light source, using blue (450–490 nm) and a 515 nm emission filter. To quantify degenerating neurons, brain sections were collected from 3.2 to 4.5 mm posterior to Bregma, and five coronal sections were randomly analyzed from each animal. A blinded observer counted the number of degenerating neurons in the hippocampus and cortex of ipsilateral hemisphere. The average number of FJB (+) neurons in each region was used for statistical analyses.

### 4.6. Detection of Oxidative Injury and Dendritic Damage

Neuronal oxidative injury and dendritic damage was measured by 4HNE and MAP2 immunofluorescence staining, respectively. Immunostaining with 4HNE and MAP2 antibodies was conducted using our previously described method [45]. Brain sections were incubated in polyclonal mouse anti-4HNE (diluted 1:500, Alpha Diagnostic Intl. Inc., San Antonio, TX, USA), or polyclonal rabbit anti-MAP2 (diluted 1:200, Abcam, Cambridge, UK) in PBS containing 0.3% Triton X-100 overnight in a 4 °C incubator. After washing three times for 10 min with 1 mM PBS, brain sections were incubated in an Alexa Fluor 488-conjugated donkey anti-rabbit IgG (MAP2), or 594-conjugated donkey anti-mouse IgG (4HNE) secondary antibody (diluted 1:250, Invitrogen, Grand Island, NY, USA) for 2 h at room temperature. The brain sections were mounted on gelatin-coated slides. 4HNE or MAP2 immunofluorescence were evaluated by an Olympus upright fluorescence microscope, and captured by a CCD cooled digital color camera (Hamamatsu Co., Bridgewater, NJ, USA) with Infinity 3 (Lumenera Co., Ottawa, ON, Canada). To quantify 4HNE or MAP2 intensity, sections were measured by a blinded experimenter using ImageJ (NIH, Bethesda, MA, USA), according to the method modified from the previously described method [46,47]. Briefly, the image was loaded into ImageJ and changed to 8 bit through the menu options (Image/Color/Split Channels). Then, the image was thresholded as follows (Image/Adjust/Threshold): the type was set to black and white and the bottom slider transferred to a value sufficient to represent solely the 4HNE or MAP2 immunoreactive area. The thresholded image was binary, and only represented 4HNE or MAP2 immunoreactivity. The selected part in the whole image was sorted. To assess this area, the menu option (Analyze/Measure) was selected, and then the signal of 4HNE or MAP2 was represented by the mean gray value.

### 4.7. Determination of Glutathione Concentration

To detect the reduced form of neuronal glutathione (GSH) concentrations, a separate group of animals were sacrificed at 24 h after TBI and brains were perfused with PFA. We then incubated brain sections at 4 °C for 4 h with a solution containing 10 mM *N*-ethylmaleimide (NEM, Sigma-Aldrich, St. Louise, MO, USA). Next, they were washed in 1 mM PBS and incubated with mouse anti-GS-NEM (GS-NEM, Millipore Co., Billerica, MA, USA, diluted 1:100). After washing, the brain sections were incubated with Alexa Fluor 488-conjugated goat anti-mouse IgG (1:400; Invitrogen, Carlsbad, CA, USA) for 2 h at room temperature. The sections were mounted on gelatin-coated slides, and fluorescence signals were detected using a Zeiss LSM 710 confocal imaging system (Carl Zeiss, Oberkochen, Germany). Stacks of images (1024 × 1024 pixels) from consecutive slices of 0.9–1.2 μm in thickness were obtained by averaging seven scans per slice, and were processed with ZEN 2010 (Carl Zeiss, Oberkochen, Germany). To measure GSH intensity, individual neurons from the images were selected as regions of interest (ROIs) and analyzed by ImageJ as our previously described methods [47,48,49]. Briefly, the image was loaded into ImageJ, and changed into an 8-bit via the menu option (Image/Type/8-bit). Then, regions involving individual neurons in the cortex and hippocampus images were selected as ROIs. The image was then binarized, and limited to the region of check for individual neurons. To assess this area, the menu option (Analyze/Measure) was selected, and then the signal from individual neurons was represented as the mean gray value.

### 4.8. Evaluation of Microglia and Macrophage Activation

To see the effects of PCA on microglial activation, PCA was administered once a day for one week. Animals were sacrificed at one week after TBI. To detect microglia and macrophage activation, three paraformaldehyde-fixed brain sections were analyzed from each brain. After washing in 1 mM PBS, sections were immunohistochemically stained with a mouse antibody-rat CD11b (diluted 1:1000, AdD Serotec, Raleigh, NC, USA) or a rabbit anti-Iba1 (diluted 1:500, Abcam, Cambridge, UK) diluted in blocking buffer (10% goat serum and 0.1% Triton X-100 in 1 mM PBS) and put overnight in a 4 °C shaking incubator. After washing, the brain sections were incubated with Alexa Fluor 488-conjugated goat anti-mouse IgG (diluted 1:250, Molecular Probes, Invitrogen) or 488-conjugated donkey anti-rabbit Ig G secondary antibody (diluted 1:250, Invitrogen, Grand Island, NY, USA) for 2 h at room temperature. To quantify the microglia and macrophage activation, the number of CD11b immunoreactive cells with continuous processes per 200 μm^2^ was counted manually from images under higher magnification (30× magnification), according to the following criteria: 0, no cells with continuously stained processes; 1, 1–9 cells with continuous processes; 2, 10–20 cells with continuous processes; 3, >20 cells with continuous processes. The morphology of CD11b immunoreactive cells was analyzed according to the following criteria: 0, 0% have activated morphology (amoeboid morphology with enlarged soma and thickened processes); 1, 1–45% of CD11b immunoreactive cells have the activated morphology; 2%, 45% to 90% of CD11b immunoreactive cells have the activated morphology; 3, >90% of CD11b immunoreactive cells have the activated morphology. The intensity of CD11b immunoreactive cells was analyzed according to the following criteria: 0, no expression; 1, weak expression; 2, average expression; 3, intense expression. Although it was hard to differentiate the cells, a well-trained, blinded observer counted it as carefully as possible. The total score was these three scores combined, ranging from 0–9 [50].

### 4.9. Detection of Live Neurons

To see the effects of PCA on delayed neuronal death, PCA was administered once a day for one week. Animals were sacrificed at one week after TBI. To evaluate whether PCA had neuroprotective effects following TBI, NeuN immunohistochemical staining was performed with brain sections. Monoclonal anti-mouse NeuN primary antibody (diluted 1:500, Billerica, Millipore Co., Billerica, MA, USA) was used at 4 °C overnight. After, the sections were washed for 10 min with PBS, incubated with biotinylated anti-mouse IgG (Vector, Burlingame, CA, USA) and ABC compounds (Burlingame, Vector, CA, USA) was diluted 1:250 in the identical liquid as the primary antiserum. Between incubations, sections were washed for 10 min with PBS. The immune response was visualized with 3,3-diaminobenzidine (DAB, Sigma–Aldrich Co., St. Louis, MO, USA) in 0.01 M PBS and the tissues were put on slides. The immune response was observed under an Olympus IX70 inverted microscope (Olympus Co., Tokyo, Japan). To quantify neuronal loss, a blinded experimenter counted the total number of NeuN (+) cells in the hippocampus and cortex as the above-described method. In addition, we used ImageJ for distinguish the difference between neurons and background noise.

### 4.10. Statistical Analysis

Data were displayed as the mean ± SEM. Statistical significance between the experimental groups were measured by analysis of variance (ANOVA) in accordance with the Bonferroni post hoc test. Difference was considered statistically significant at *p* < 0.05.
